# Report on the Etiological and Pathological Relations of Epidemic Erysipelas, Spotted Fever, and Diphtheria

**Published:** 1867-02

**Authors:** N. S. Davis

**Affiliations:** Prof. of Principles and Practice of Medicine and of Clinical Medicine, in Chicago Medical College


### Selections.

### REPORT ON THE ETIOLOGICAL AND PATHOLOGICAL RELATIONS OF EPIDEMIC ERYSIPELAS, SPOTTED FEVER, AND DIPHTHERIA.

By N. S. DAVIS, M.D., Prof. of Principles and Practice of Medicine and of Clinical Medicine, in Chicago Medical College.

[From the Transactions of the American Medical Association.]

There is no department of medical study environed with greater difficulties than that of etiology. And especially is this true of the etiology of such diseases as are styled epidemic. They appear at uncertain, and often remote, intervals; in sections of country widely diverse in relation to geological formation, climate, season, etc.; attack large numbers in quick succession; and then disappear almost as rapidly as they were developed. The elements and agents, mental and physical, ponderable and imponderable, that are continuously surrounding and capable of impressing an influence upon every living human being, are so numerous and capable of such varied combinations that the problems presented for solution in the study of the causes of disease are among the most complex and obscure that can engage our attention. To solve these problems successfully requires a complete record of the electrical, barometrical, thermometrical, hygrometrical, and ozonic conditions of the atmosphere for a series of years; the position and composition of the soil; the quality of the water; the individual and social habits of the people in relation to dress, diet, drinks, structure of houses, and habits of thought, in direct connection with a faithful account of the kind, amount, and specific character of all the diseases that may occur, whether they are epidemic or not.

At present, so far as relates to our country, these records do not exist, except to a very limited extent, and hence the data essential for determining with accuracy the causes of any particular epidemic do not exist. To commence systematically, the work of patient and faithful observation and record of facts in relation to all the topics just named, is one of the most important steps that can be taken for the further promotion of medical science. It is easy to assume the existence of some special and subtle poison as the cause of any given epidemic form of disease, and almost equally easy to enter upon extensive and plausible speculations in relation to its effects. While to demonstrate the existence or non-existence of such a poison in connection with the prevalence of any given disease may require the patient and persistent use of all the means furnished by modern science, through a series of years. It is not surprising, therefore, that our literature should be filled with an almost endless amount of speculation in regard to fever, cholera, and blood or zymotic poisons, until the intelligent student can hardly fail to be reminded of the concoctions, fermentations, and humors of a more remote period in the history of medical science. It may not be proper to claim that every specific morbific agent or poison should be positively identified and its properties determined before its existence can be logically admitted; because certain effects may be found to follow so uniformly the coëxistence of certain circumstances, that the development of an active agent, capable of producing certain appreciable effects, may be a fair and legitimate conclusion deduced from the premises. But if we would reduce the uncertainties of medical science, lessen the number of vague, intan-

6

gible and contradictory generalizations in medical literature, and substitute therefor real advancement in medical science and art, we must begin by patiently recording observed facts instead of *opinions*, and rigid inductive conclusions instead of mere *assumptions.* And if that section of the American Medical Association, having charge of the subjects of meteorology, medical topography, and epidemic diseases, could devise and carry into effect a successful plan for accomplishing this change so far as relates to those departments of medical science, it would constitute a more important step for the advancement of etiology and hygiene than has been taken during the past half century.

It is certain, however, that so long as mere opinions are allowed to supply the place of facts, and every writer or teacher is permitted to assume the existence of any specific poison that he can make subservient to his theories, our literature will continue to be filled with the most discordant opinions and the most vague and diverse theories. We shall continue, as heretofore, to amuse and bewilder ourselves with speculations as contrary to the uniformity of nature's laws, as they are to the acknowledged rules of inductive philosophy. One of the most prolific sources of error in our profession consists in partial or incomplete investigations, and the record of only a part of the facts relating to any given subject. This becomes speedily apparent to every one who institutes a serious inquiry after the cause or causes of any particular form of disease. He will find abundant announcements that the disease made its appearance in a given community at such a time; attacked such a number, chiefly of such a class; continued more or less severe for such a time; and disappeared. Not unfrequently these general statements are accompanied by equally general allusions to some one prominent sanitary, hygienic, or meteorological circumstance occurring coïncidently with the disease. But how few are the instances, in the whole range of our voluminous literature, in which the account of any single epidemic prevalence of disease is accompanied by so full a statement of the preceding and accompanying topographical, meteorological, and hygienic conditions, including the special personal hygienie, physical and mental, of those attacked, that we can form a full and reliable idea of the influences that were brought to bear upon the properties and functions of living beings. And yet, without this completeness of facts, all etiological deductions must be more liable to be erroneous than truthful. It is probable that a careful review of the history of epidemic diseases would show them capable of being arranged etiologically into two classes. The first class embraces all such as arise directly from a specific virus or animal poison capable of being reproduced in the living animal body, and capable of being propagated in any climate, at any season of the year, and without regard to public or individual hygienic conditions. These, however much they may be influenced in their severity and fatality by collateral circumstances, are universally admitted to depend essentially upon a class of specific poisons, and hence are indisputably contagious.

The second class embraces such diseases as appear in an epidemic form only at irregular intervals; prevail only for a limited period of time; and at any given prevalence, appear incapable of propagation beyond certain geographical limits or sanitary influences. To this class belong influenza, spotted-fever, erysipelas, diphtheria, cholera, etc.

The fact that these diseases have appeared successively or at different times in almost every part of the world; at every season of the year; and among widely different classes of people, has caused their essential causes to be involved in great obscurity, and to become the theme of almost endless speculations and controversies. That they are not like endemics, dependent on causes originating in any particular soil or geological formation, is fully apparent from their occurence in localities so widely diverse in this respect.

The most popular theory is, that a part, at least, of the diseases enumerated in this class arise essentially from some infectious poison originating from some primary focus, and spreading from there, chiefly through the atmosphere, but capable of transportation, also, in the holds of ships, in boxes of goods, etc., to the most distant parts of the civilized world. Hence, the common expressions applied to them are, they are coming, going, or traveling. While it is generally acknowledged that local sanitary conditions exert a controlling influence over the extent and severity of any one or all of this class of diseases, yet it is claimed that they are incapable of originating them. The popular idea, however, that a pestilential poison, too subtle for identification and scrutiny, can originate at some focal point, and from thence extend from place to place around the globe, will scarcely bear the test of a rigid investigation. All the laws of chemical or physical science indicate that for the production of any inorganic body, whether solid, fluid, or gaseous, the elements entering into its composition and the circumstances under which they unite, are uniform. Again, all that has yet been ascertained in regard to the development of vegetable fungi or sporules and animalculæ, indicates that each species of these minute organizations requires fixed local conditions for their development, and a very limited meteorological and geographical range in which they are capable of self-propagation.

To find a species of animalcule generated on the banks of the Ganges and propagating itself on the snows of Northern Russia, would be as much an anomaly in nature as to find the lion of tropical Africa flourishing in the arctic regions of North America. It is very generally alleged that each of the diseases in the class under consideration is identically the same wherever it occurs, and hence there must be an identity or specific character to the cause. If we grant this position, it simply necessitates the assumption of another, namely, that the production and propagation of an identical specific cause necessitates the presence of certain uniform materials and circumstances out of which that cause can be evolved. When we find a disease limited in its prevalence to certain geographical and geological boundaries, we naturally look to the soil as the essential source from which the cause of such disease is capable of being produced; because the composition and conditions of the soil are the uniform circumstances peculiar to the localities in question.

So too, when we find a disease identical in its character prevailing on many different geological formations, in various climates, and at different seasons of the year, the same rules of induction would require us to look for the origin of its cause in some materials and circumstances that are common to all the localities in which the disease itself prevails. And if we find man himself and the conditions of social life the only conditions uniformly present, logically we must look to these as furnishing both the materials and the conditions for evolving the immediate cause of the disease. There is no reason why diseases of a specific character and epidemic prevalence may not be engendered by disturbance of vital properties either with or without retention of certain excrementitious materials, as well as by the introduction of special poisons or miasms from without. Indeed, there are so many points of striking contrast between the first class of epidemics known to arise directly from the introduction of an animal poison capable of reproduction and propagation in the living body, and those constituting the second class, as to afford strong evidence of the entire dissimilarity of their causes.

Each individual disease of the first class has its definite period of incubation after the reception of the poison; an equally definite period of irritative fever before the local inflammations or eruptions are established; with an almost uniform period of progress, maturity, and decline of such local manifestations, thus making each case a fixed order of events, each occupying a self-limited time, and always accompanied by the evolution or reproduction of more or less of the same specific poison that had induced the disease.

It is hardly necessary to add, that no such order of events, definite as to time and duration, has been observed in influenza, cholera, diphtheria, erysipelas, or spotted-fever; and certainly no evidence of any uniform evolution of a specific poison during their progress. If, under some circumstances, the secretions and emanations from the bodies of patients sick with any one of these diseases, have appeared to be capable of communicating the disease directly to others, they have constituted rare exceptions to the general rule.

All the diseases of the first class are self-protective, except in very rare instances, occurring only once in the same individual. While the reverse is true of all those belonging to the second class.

Again, the diseases of the first class never *relapse*. Those of the second often.

There is still another point of difference having an important bearing upon the etiology of the diseases under consideration. It is, that all the diseases of the second class have their analogous or typical forms, among the ordinary sporadic diseases of each returning season. Thus, influenza has its type in the severer cases of coryza or acute catarrh; cholera, in the cholera-morbus of every returning summer; diphtheria, in follicular and erythematic inflammations of the fauces; and erysipelas, in the sporadic and traumatic cases of common occurrence.

Inasmuch as all these diseases in their sporadic forms or types are everywhere acknowledged to arise from strictly local meteorological and sanitary conditions, and equally conceded that in their epidemic forms they are greatly influenced both in numerical prevalence and fatality, by the same causes, would it not be more philosophical as well as more in consonance with observed facts, to attribute the epidemic form of any one of them to a simple exaggeration or modification of the same causes that give rise to sporadic cases, without calling to our aid imaginary poisons, and assuming for them powers of diffusion contrary to all the known laws of organic or inorganic matter.

These general observations have been pursued much farther than was originally intended; but they seemed necessary as an introduction to the views I wished to present in relation to the special causes of erysipelas, spotted-fever or cerebro-spinal meningitis, and diphtheria. With the exception of influenza and cholera, no diseases have prevailed more extensively as epidemics than the three just named. They are almost universally regarded by the profession as blood diseases, or, in other words, as dependent upon primary morbid conditions of the blood, inducing secondary local inflammations in various parts of the body. A careful study of the phenomena and results of these diseases, has led me to doubt whether the morbid conditions of the blood are really primary and dependent on the introduction into it of some specific poisons from without, or whether they are the effects of a prior disturbance or perversion of those vital affinities by which the primary steps in the processes of disintegration and secretion are performed, thereby leading either to the evolution of some morbid product to be returned into the blood, or the failure to evolve some natural product which by its absence would no less certainly leave that fluid in a morbid condition. That the blood presents an unnatural or diseased condition while the system is affected by either of the diseases under consideration none will deny. But whether such change in the blood constitutes the primary or essential pathological condition on which all the subsequent phenomena and results of the disease depend, or are only a result of prior changes in the properties and functions of the organized structures is a question of more than merely speculative importance. Ever since the prominence given to pathological anatomy by the French school of which Louis, Chomel, Andral, etc., were such noble ornaments, there has been a manifest tendency to confound morbid anatomy or the effects of disease with true pathology. The investigations necessary to enable me to give a positive answer to the questions just stated, have not yet been completed. But thus far, they have led me to believe that the essential causes of erysipelas are such as are capable of directly depressing and, to some extent, perverting the elementary vital affinities of the tissues, in such a direction as to disturb the processes of disintegration and to diminish the excretion of alkaline salts. This induces, speedily, an excessively alkaline condition of the blood coïncident with the accumulation of the products of a disturbed tissue metamorphosis or disintegration. The circumstances capable of producing these primary pathological changes are various, but chiefly involve alterations in the electrical and ozonic conditions of the atmosphere, often rendered more efficient by an excess of carbonic acid gas and aqueous vapor. Every practical surgeon is familiar with the fact, that if he places an undue number of wounded patients in a confined atmosphere, their wounds speedily assume an unhealthy condition, leading directly to erysipelas or gangrene; while if the same patients are left in the open air, sheltered only by tents, no such effects follow. Now it can readily be demonstrated that such confinement of the air with an accumulation of exhaled carbonic acid gas and aqueous vapor is capable of changing its ozonic and electrical conditions, even in a few hours. It is also generally conceded that these atmospheric constituents are capable of undergoing sudden and important changes over extensive districts of country, regardless of geological formations or parallels of latitude; thus affording an adequate explanation of the occurrence of the disease in localities so various, and with such variable degrees of severity. This view of the essential pathology and causation of erysipelas is corroborated by the well-known fact that the habitual use of drinks which, like alcohol, retard the natural tissue changes, decidedly predispose to attacks of erysipelas or, in other words, favor the development of the erysipelatous diathesis.

The conclusion at which I have arrived in regard to the disease promiscuously styled "spotted fever" and "cerebro-spinal meningitis," is, that as reported in our literature, no less than three or four distinct diseases have been confounded together.

The first class of cases, embracing those most extensively prevalent as well-marked epidemics, and tending most rapidly to fatal results, were no more nor less than true erysipelas, in which the cerebro-spinal nervous structures with their enveloping membranes, constituted the seat of the local inflammatory developments instead of the fauces or cutaneous surfaces. This view is sustained by the immediate preceding or coïncident prevalence of well-marked cutaneous erysipelas, which has been noticed by most of the later writers on this subject; by the rapid progress of the disease; by the almost uniform aplastic character of the local exudations and changes; and by the fact of cases in which manifest erysipelas of the fauces and face has been accompanied by all the pathognomonic symptoms of fatal cerebro-spinal meningitis. One case of this kind occurred under my own observation only a few months since. In a recent paper on the cerebro-spinal meningitis that prevailed so severely and extensively in the middle and western states between 1844 and 1848, read to the Illinois State Medical Society, Dr. J. ADAMS ALLEN, formerly in the medical department of University of Michigan, says: "Erysipelas is the most frequent and dangerous of complications. It is easily kindled upon the slightest abrasion of the surface, and often, without such a nidus, attacks the face and neck, extending to the scalp, or more rarely seizing the extremities."

Dr. J. S. JEWELL, of the Chicago Medical College, in a letter presented to the same society, speaks of witnessing an epidemic both of "spotted fever or cerebro-spinal meningitis" and of "erysipelas" in the same localities in the southern part of Illinois during the years 1862–3. Close examination of another class of cases will show them to be either modified forms of typhus or of scarlatina. While still another class, much fewer in number, and generally occurring sporadically, are simple acute inflammations of the membranes enveloping the cerebro-spinal axis and base of the brain.

In regard to diphtheria, I will only add to the observations already made, the opinion that its causative influences are analagous to, though not identical with, those of erysipelas. That while they induce such modifications of vital affinity in the tissues generally as to greatly lessen their tonicity, and such disturbances of the atomic or cell changes of tissue metamorphosis as to cause a morbid condition of the effete elements of the blood, they also produce a well marked tendency to aplastic albuminous exudations, as shown by the rapid formation of aplastic deposits on all inflamed or abraded surfaces and the elimination of albumen through the kidneys. These changes in the properties of the elementary structures and in the nitrogenous elements of the blood would necessarily develope great debility with so much spanemia or blood impoverishment, as to afford a rational explanation of such well-known and peculiar sequelæ of this disease, as unusual muscular debility, protracted convalescence, and frequent paralysis, either partial or complete. I have thus compressed, into less than a dozen pages of manuscript, an outline of those views of the etiology and pathology of erysipelas, spotted fever, and diphtheria which would require the compass of a volume to illustrate the facts, which might be adduced for the purpose of testing their claim to credibility. My sole object in presenting them, is to elicit discussion and excite more rigid and extensive investigations.

